# Tailoring Charge Donor–Acceptor Interaction in CsPbBr_3_ Perovskite Nanocrystals through Ligand Exchange

**DOI:** 10.1002/smsc.202300348

**Published:** 2024-03-26

**Authors:** Syed Abdul Basit Shah, Sushant Ghimire, Rostyslav Lesyuk, Maria Vittoria Diamanti, Vanni Lughi, Christian Klinke

**Affiliations:** ^1^ Department of Engineering and Architecture Università degli studi di Trieste Trieste 34127 Italy; ^2^ Institute of Physics University of Rostock 18059 Rostock Germany; ^3^ Pidstryhach Institute for Applied Problems of Mechanics and Mathematics of NAS of Ukraine Naukowa 3b Lviv 79060 Ukraine; ^4^ Department of Chemistry, Materials and Chemical Engineering “Giulio Natta” Politecnico di Milano Milan 20131 Italy; ^5^ Department of Chemistry Swansea University Swansea SA2 8PP UK; ^6^ Department “Life, Light & Matter” University of Rostock 18059 Rostock Germany; ^7^ Present address: Chair for Photonics and Optoelectronics Nano‐Institute Munich and Department of Physics Ludwig‐Maximilians‐Universität (LMU) 80539 Munich Germany

**Keywords:** charge transfer, donor–acceptor interactions, ligand exchange, metal halide perovskites, mixed photoluminescence quenching, quenching sphere of action

## Abstract

The surface ligands in colloidal metal halide perovskites influence not only their intrinsic optoelectronic properties but also their interaction with other materials and molecules. Donor–acceptor interactions of CsPbBr_3_ perovskite nanocrystals with TiO_2_ nanoparticles and nanotubes are explored by replacing long‐chain oleylamine ligands with short‐chain butylamines. Through postsynthesis ligand exchange, the nanocrystals are functionalized with butylamine ligands while their intrinsic properties are maintained. In solution, butylamine‐capped nanocrystals exhibit reduced photoluminescence intensity with increasing TiO_2_ concentration but without any change in photoluminescence lifetime. Intriguingly, the Stern–Volmer plot depicts different slopes at low and high TiO_2_ concentrations, suggesting donor‐acceptor interaction through mixed static photoluminescence quenching and quenching sphere of action mechanism . Oleylamine‐capped nanocrystals in solution, on the other hand, show no interaction with TiO_2_, as indicated by consistent photoluminescence intensities and lifetimes before and after TiO_2_ addition. In films, both types exhibit decreased photoluminescence lifetime with TiO_2_, indicating enhanced donor–acceptor interaction, which is discussed in terms of electron transfer. TiO_2_ nanotubes enhance nonradiative recombination more in butylamine‐capped CsPbBr_3_ perovskite nanocrystals, emphasizing the role of ligand chain length.

## Graphical abstract

Enhanced electronic interaction between cesium lead bromide (CsPbBr_3_) perovskite nanocrystals (PNCs) as electron donors and titanium oxide (TiO_2_) as electron acceptors in solution and film is demonstrated by substituting the long‐chain oleylamine ligand with a shorter‐chain butylamine on the PNC surface post‐synthetically. This research provides a significant understanding of perovskite‐based heterojunctions, enabling efficient charge transfer for effective charge carrier harvesting.

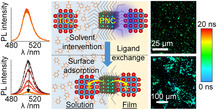

## 1 Introduction

3D ABX_3_ perovskites are outstanding semiconductors due to their excellent optoelectronic properties and diverse applications, including photovoltaics, LEDs, photodetectors, lasers, and photocatalysis.^[^ ^1^ ^]^ Here, A is a monovalent cation such as Cs^+^, methylammonium (MA), or formamidinium (FA) occupying a cavity formed by the corner‐sharing (BX_6_)^4−^ octahedra consisting of a divalent metal ion B^2+^ (B = Pb, Sn, Ge) and halide ions X^−^ (X = Cl, Br, I). Among the various compositions of metal halide perovskites, all‐inorganic perovskite nanocrystals (PNCs) have gained the spotlight on account of their notable stability, nearly unity photoluminescence (PL) quantum yield (QY), and compatibility with optoelectronic devices.^[^ ^2^ ^]^ Manipulating the surface of these PNCs through ligand engineering is necessary to optimize their functionality and expand their applications.

Surface ligands play a crucial role in regulating not only the shape and the size of ABX_3_ PNCs^[^ ^3^ ^]^ but also their optoelectronic properties, stability, and surface chemistry.^[^ ^2^, ^4^ ^]^ Surface passivation of PNCs by ligands stabilizes the intrinsic defects as well as prevents molecules such as water and oxygen from reaching their surfaces. This, on the one hand, suppresses the nonradiative decay channels for excitons and hence improves the PL QY and charge carrier dynamics of PNCs, and, on the other hand, enhances their intrinsic as well as their environmental stability. While oleic acid and oleylamine are the conventionally used combination of Lewis acid–Lewis base‐based binary ligands for synthesizing shape‐ and size‐controlled, stable, and brilliantly luminescent PNCs and quantum dots (QDs),^[^ ^5^ ^]^ other ligands including phosphine/phosphine oxide,^[^ ^6^ ^]^ sulfonate,^[^ ^7^ ^]^ quarternary ammonium,^[^ ^8^ ^]^ and zwitterionic or bidentate ligands[^2^, ^9^] are also used either in combination with the above ones or independently to realize high‐quality nanocrystals. In all cases, the role of surface ligands in stabilizing and improving the properties of ABX_3_ PNCs is crucial.

Besides shape and size control, defect passivation, and stability, the role of surface ligands is important when considering the photophysical interaction of PNCs among themselves and/or with other semiconductor materials, metals, and molecules that may result in a charge or energy transfer.^[^ ^10^ ^]^ The presence of bulky surface ligands may act as an insulating layer for charge transfer between PNCs and other charge‐accepting/donating species. This greatly influences the efficiency of devices where ABX_3_ PNCs are used as light‐absorbing or light‐emitting layers.^[^ ^4^, ^11^ ^]^ Also, such ligands may limit the use of PNCs in photocatalysis where exciton dissociation and charge separation are imperative to conduct heterogeneous photoredox reactions.^[10g]^ On the other hand, the removal of excess ligands from the PNC surface may improve the charge transfer process but compromise the stability and optoelectronic properties.^[^ ^12^ ^]^ Therefore, a balance is required for controlled charge transfer processes in PNCs with minimal influence on their stability and properties.

The use of certain aromatic or conjugated ligands in the synthesis of metal halide perovskites can promote charge delocalization.[^10^, ^13^] Vickers et al. demonstrated that the film of MAPbBr_3_ QDs synthesized using benzylamine and benzoic acid ligands showed superior electrical conductivity, extended carrier lifetime, and effective charge transfer to a fluorine‐doped tin oxide glass substrate, as opposed to QDs with nonconductive ligands.^[10a]^ Also, passivation of the ABX_3_ PNC surface with short‐chain ligands during the synthesis can facilitate better electronic coupling among PNCs or with other charge donor/acceptor species, enhancing the efficiency of charge transfer.[^10^, ^12^] Kumar et al. showed an increase in current density and luminance and a decrease in turn‐on voltage of an FA_0.5_MA_0.5_PbBr_3_‐based LED while decreasing the chain length of the aliphatic amine ligand from 16 to 6 carbon.^[10c]^ They attributed the effect of ligand chain length on the enhanced electroluminescence characteristics of FA_0.5_MA_0.5_PbBr_3_ PNC film to an accelerated rate of charge transfer between nanocrystals, which further enhanced charge injection into the PNC layer within the LED device.

Controlling the morphology of 3D ABX_3_ PNCs through the use of conjugated/aromatic ligands or short‐chain ligands during synthesis can be a complex task. Short‐chain ligands, for example, may lead to the formation of quasi‐2D perovskite nanoplatelets or a mixture of nanoplatelets and nanocubes.^[^ ^3^, ^14^ ^]^ Similarly, employing short‐chain, conjugated, or aromatic ligands might also result in the development of layered 2D Ruddlesden–Popper structures instead of the desired 3D perovskite nanocubes.^[^ ^15^ ^]^ These low‐dimensional metal halide structures hold promise for optoelectronic properties, albeit differing from those of 3D ABX_3_ PNCs.^[^ ^16^ ^]^ This disparity makes it challenging to directly compare the charge transfer process in long‐ and short‐chain ligand‐capped PNCs and metal halide nanostructures. Such a comparison is crucial for a deeper understanding of photophysics in charge donor–acceptor systems based on 3D ABX_3_ PNCs and is essential for their effective utilization in light‐emitting and light‐harvesting applications. Postsynthesis ligand exchange, on the other hand, could be an effective strategy for manipulating the surface of ABX_3_ PNCs for efficient charge transfer while preserving their morphology and properties.^[^ ^4^, ^17^ ^]^

Previous studies discussed different ligand binding modes on the ABX_3_ PNC surface, suggesting that the organic acid and organic amine ligands are weakly attached to the PNCs and exhibit highly dynamic interactions with the PNC surface.^[^ ^4^, ^18^ ^]^ This allows for easy ligand exchange, with the native ligands on the surface of PNCs being replaced with the new desired ones. As a result, charge transfer from PNCs to other semiconductors or molecules can be enhanced by precisely tuning the surface ligand length through postsynthesis ligand exchange.[^10^, ^19^] Biswas et al. successfully demonstrated the postsynthesis exchange of oleic acid in CsPbBr_3_ PNCs with short‐chain benzoic acid and ascorbic acid ligands.^[10d]^ While both the ligand‐exchanged samples showed improved stability and enhanced optoelectronic properties, the benzoic acid ligand‐capped CsPbBr_3_ PNCs exhibited better charge transfer rates to the acceptor molecules such as *para*‐benzoquinone (electron acceptor) and phenothiazine (hole acceptor) due to the conjugated and short ligand chain. Similarly, Dey et al. showed improved electronic coupling, and therefore enhanced charge transfer across the CsPbBr_3_ PNC–CdSe nanoplatelet heterojunction which is cross‐linked via *para*‐aminobenzoic acid or glycine ligands postsynthetically through ligand exchange when compared with their mixture with native long‐chain oleic acid and oleylamine ligands.^[^ ^19^ ^]^

In this study, we highlight the enhanced interaction between CsPbBr_3_ PNCs as electron donors and TiO_2_ nanostructures as electron acceptors, achieved by substituting the long‐chain oleylamine ligand with a shorter chain butylamine on the PNC surface postsynthetically. We chose TiO_2_ as an electron acceptor owing to its type‐II band alignment with metal halide perovskites.^[^ ^20^ ^]^ TiO_2_ has been extensively studied as a thermally and chemically stable, low‐cost, electron transport layer with suitable band‐edge positions for perovskite solar cells as well as active photocatalyst for chemical conversions and water splitting.[^1^, ^21^] We explore aspects such as PL quenching, electron‐transfer dynamics, and trap‐state modifications resulting from the donor–acceptor interaction. In solution, only butylamine (ButAm)‐capped CsPbBr_3_ PNCs demonstrate interactions with TiO_2_, resulting in noticeable PL quenching. Conversely, in film states, both ButAm‐ and oleylamine (OlAm)‐capped PNCs exhibit robust donor–acceptor interactions with TiO_2_. The interaction is significantly amplified in CsPbBr_3_ PNCs with the shorter chain butylamine ligands, emphasizing efficient electron transfer to TiO_2_. This study presents pivotal insights into fine‐tuning the PNC–TiO_2_ donor–acceptor interactions through postsynthesis ligand exchange. Further exploration of this approach holds promise in engineering metal halide perovskite‐based heterojunctions with efficient charge transfer, benefiting perovskite solar cells, LEDs, and photocatalysis.

## 2 Results and Discussion

**Figure 1:**
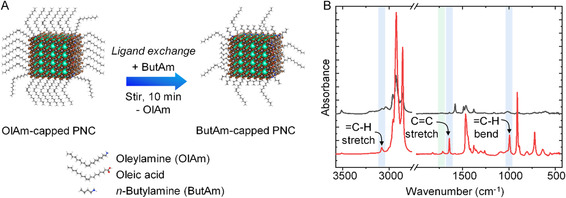
A) Scheme showing the exchange of long‐chain oleylamine ligands on the surface of PNCs with short‐chain butylamine ligands. B) FTIR spectra of CsPbBr_3_ PNCs before (red) and after (black) ligand exchange. The light blue regions correspond to the vibration frequencies of oleylamine, and the light green region corresponds to the vibration frequency of oleic acid.

We investigated how altering the chain length of aliphatic amine ligands impacts the CsPbBr_3_ PNC–TiO_2_ donor–acceptor interactions in both solution and film states. We employed a postsynthesis ligand exchange approach to functionalize the PNC surface with short‐chain butylamine ligands. Initially, we synthesized CsPbBr_3_ PNCs using a hot‐injection method reported earlier by Protessescu et al. with slight modifications.^[5a]^ Oleic acid and oleylamine were used as capping ligands for the PNCs. The as‐synthesized PNCs were directly subjected to ligand exchange without any additional washing steps. **Figure** **1**A shows the scheme for postsynthesis ligand exchange on as‐synthesized CsPbBr_3_ PNCs. The ligand exchange was conducted under ambient conditions by introducing butylamine to the OlAm‐capped PNCs in toluene while stirring at room temperature. We optimized the amount of butylamine to 2 μL (20 μmol) per 3 mL (6 μmol) of PNC solution during the ligand exchange without causing damage to the original OlAm‐capped PNCs. Indeed, we observed that adding a significant amount of butylamine (≥10 μL) to the same amount of OlAm‐capped CsPbBr_3_ PNCs causes a noticeable change in the color of the colloidal solution, shifting from green to colorless and quenching of PL (Figure S1, Supporting Information). Previous studies have highlighted a similar effect concerning the ligand‐assisted degradation of PNCs or their transformation to other phases.[^5^, ^22^] After the ligand exchange, the sample was washed thoroughly by adding acetonitrile to remove excess ligands, and the resulting ButAm‐capped PNCs were collected by centrifugation and redispersed in toluene for further studies. We used Fourier transform infrared (FTIR) spectroscopy in attenuated total reflection mode to confirm the ligand exchange process. Figure 1B provides a comparison of the FTIR spectra of OlAm‐capped CsPbBr_3_ PNCs with those of ButAm‐capped PNCs after the ligand exchange. Following the ligand exchange, IR absorption bands at approximately 3080, 1642, and 990 cm^−1^ that are related to =C—H stretching, C=C stretching, and =C—H bending vibrations, respectively,^[^ ^23^ ^]^ and are closely associated with oleylamine or oleic acid showed a significant reduction or disappearance in ButAm‐capped PNCs. Notably, the IR band at approximately 1715 cm^−1^, associated with the C=O stretching vibration of an organic acid,^[^ ^23^ ^]^ also diminished in ButAm‐capped PNCs compared to the OlAm‐capped ones. These findings indicate that during the ligand exchange, butylamine largely replaces oleylamine on the surface of CsPbBr_3_ PNCs. Concurrently, the concentration of oleic acid ligands on the PNC surface also decreases. These results align with the dynamic ligand binding mode, where oleate binds to the PNC surface as an ion pair with oleylammonium.^[18a]^ Hence, replacing oleylamine with butylamine on the PNC surface results in the removal of oleic acid, reducing its concentration on the PNC surface.

**Figure 2:**
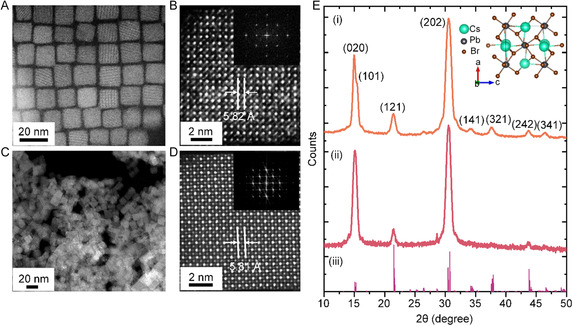
A,C) STEM and B,D) HR‐STEM images of as‐synthesized OlAm‐capped (A,B) and ligand‐exchanged ButAm‐capped (C,D) CsPbBr_3_ PNCs. The insets in (B,D) are the corresponding FFT images. E) Powder XRD patterns of (i) ButAm‐capped and (ii) OlAm‐capped CsPbBr_3_ PNCs which are compared with (iii) ref. [26]. The inset shows the crystal structure of orthorhombic CsPbBr_3_.

We examined the morphology and crystal structure of OlAm‐ and ButAm‐capped CsPbBr_3_ PNCs using high‐resolution scanning transmission electron microscopy (HR‐STEM) and powder X‐ray diffraction (XRD), as detailed in **Figure** **2**. The STEM images show that the OlAm‐capped PNCs are cube‐shaped with an average size of 12 nm (Figure 2A and S2A, Supporting Information) which is maintained after ligand exchange with butylamine (Figure 2C and S2B, Supporting Information). Conversely, the ButAm‐capped CsPbBr_3_ PNCs exhibited aggregation on the TEM grid due to reduced interparticle distance caused by the shorter chain ligands on the PNC surface. Further, the HR‐STEM image of a ButAm‐capped CsPbBr_3_ PNC in Figure 2D displays a lattice spacing of 5.81 Å, consistent with that of an OlAm‐capped PNC (Figure 2B) and corresponds to the (101) plane of an orthorhombic CsPbBr_3_ crystal structure.^[^ ^24^ ^]^ This is further supported by the powder XRD patterns of OlAm‐ and ButAm‐capped CsPbBr_3_ PNCs shown in Figure 2E. In the XRD pattern, the diffraction peak at a 2*θ* angle of 15° in OlAm‐capped CsPbBr_3_ PNC (Figure 2E,i) split into (020) and (101) peaks, corresponding to the orthorhombic crystal phase.^[^ ^25^ ^]^ Nevertheless, the broadening of XRD peaks hinders the clear observation of peak splitting for both samples when compared with the bulk CsPbBr_3_, which is attributed to the small crystal size.^[^ ^26^ ^]^ Still, all the diffraction peaks in both cases align well with those of the orthorhombic CsPbBr_3_ reported in the literature.^[^ ^25^ ^]^

**Figure 3:**
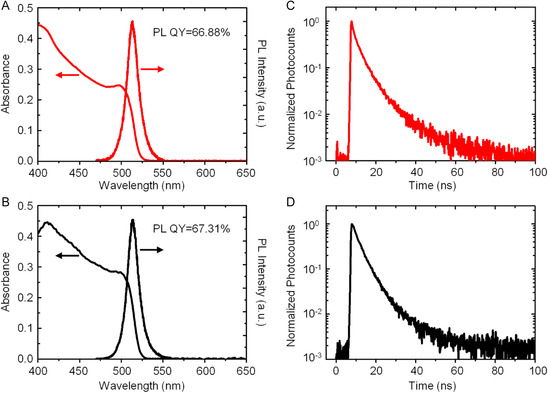
A,B) Absorption and PL spectra of OlAm‐capped (A) and ButAm‐capped (B) CsPbBr_3_ PNCs. C,D) PL decay profiles of OlAm‐capped (C) and ButAm‐capped (D) CsPbBr_3_ PNCs.

We characterized the optical and PL properties of CsPbBr_3_ PNCs in solution before and after the ligand exchange using absorption, steady‐state, and time‐resolved PL spectroscopy. **Figure** **3**A,B shows absorption and PL spectra for OlAm‐ and ButAm‐capped PNCs, respectively, showcasing comparable results. The spectra display the absorption onset at 528 nm with the near‐band‐edge exciton absorption occurring at 500 nm. The PL peak is observed at 510 nm, featuring a full width at half maximum (FWHM) of 18 nm. Further, both the samples exhibit bright green emission with an absolute PL QY of 67% (Figure S3, Supporting Information). To understand the exciton recombination dynamics, we recorded the PL decay profiles for OlAm‐ and ButAm‐capped CsPbBr_3_ PNC solutions using a time‐correlated single‐photon counting (TCSPC) system, which are shown in Figure 3C,D, respectively. We analyzed the decays by fitting them with a triexponential decay function and calculated the intensity‐weighted average PL lifetimes (*τ*). The obtained *τ* values are 5.96 ns for OlAm‐capped PNCs and 5.50 ns for ButAm‐capped PNCs, demonstrating similar PL lifetime in both cases. These findings affirm that the CsPbBr_3_ PNCs maintain their optical and PL properties after undergoing postsynthesis ligand exchange.

**Figure 4:**
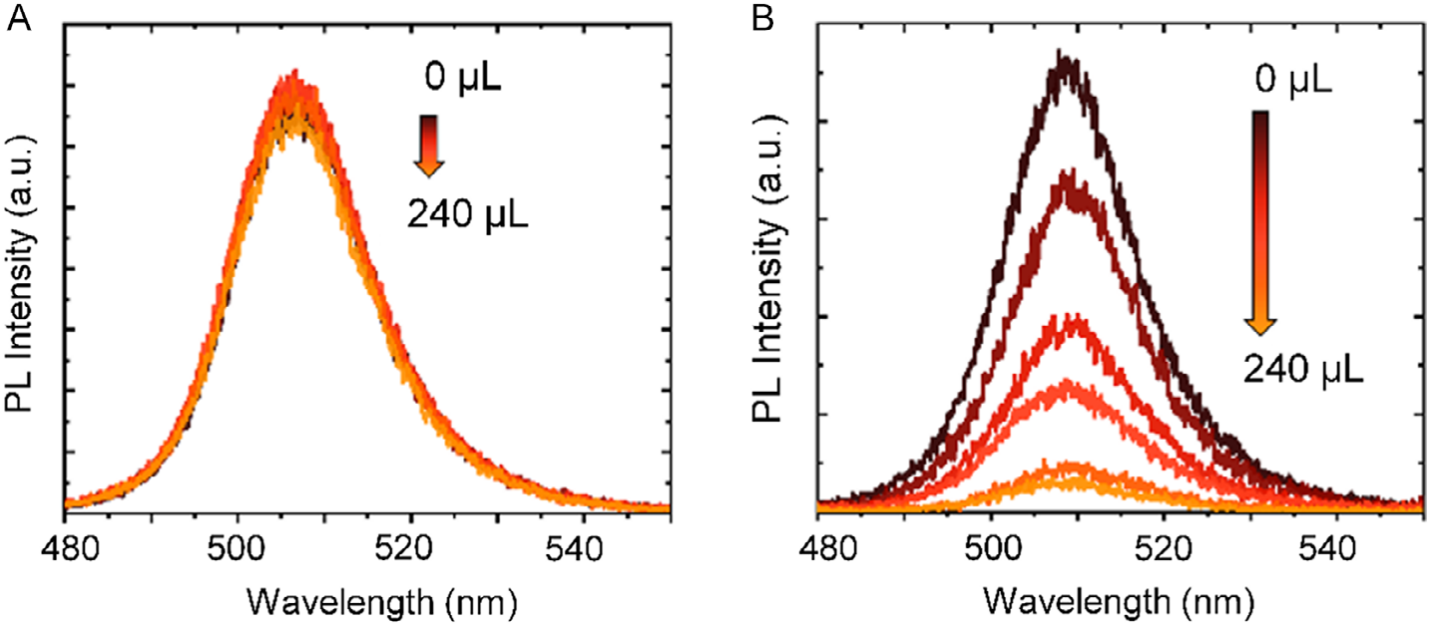
A,B) PL spectra showing PL quenching of CsPbBr_3_ PNCs by TiO_2_ in solution before (A) and after (B) ligand exchange.

With successful postsynthesis ligand exchange that maintains the morphology, crystal structure, optical absorption and PL properties of CsPbBr_3_ PNCs, we investigated the influence of ligand chain length on the donor–acceptor interactions between PNCs and TiO_2_ nanoparticles (NPs) and nanotubes (NTs) in the solution and film state. In the solution phase, we began by examining the PL spectra of OlAm‐ and ButAm‐capped PNCs with the systematic addition of different volumes of TiO_2_ suspension in toluene. The colloidal PNC solutions for both samples were suitably diluted to an optical density of 0.5 to ensure a comparable nanocrystal concentration and mitigate any inner filtering effects. To these solutions, we added TiO_2_ NP (size 50–150 nm, Figure S4A, Supporting Information) suspension at different volumes increasing from 0 to 240 μL, corresponding to final concentrations ranging from 0 to 120 mM. The stock suspension was prepared by mixing 1 g of TiO_2_ NPs in 14 mL of toluene. The structural and optical characterization of TiO_2_ NPs are provided in Figure S5, Supporting Information. As shown in **Figure** **4**A, we observed constant PL intensities for the OlAm‐capped CsPbBr_3_ PNC solution, displaying no PL quenching in the presence of TiO_2_. However, in the case of the ButAm‐capped PNC solution, the PL intensities decreased (Figure 4B) with an increase in the added volume of TiO_2_ suspension. Here, the PL spectra obtained after each addition of TiO_2_ suspension in toluene are corrected for the dilution effect, the details of which are provided in the Supporting Information. Also, the PNC solutions were colloidally stable at different dilutions as indicated by the consistent absorption spectra of CsPbBr_3_ PNCs at different volumes of TiO_2_ suspension added as shown in Figure S6, Supporting Information. These results suggest that the short‐chain ligands enhance the photophysical interactions between the charge donor and acceptor species. Considering the type‐II band alignment between CsPbBr_3_ and TiO_2_,^[^ ^20^ ^]^ we anticipate that the donor–acceptor interactions in our case involve electron transfer. Nevertheless, to understand this further, one has to look closer into the dynamic aspects of the quenching interaction.

**Figure 5:**
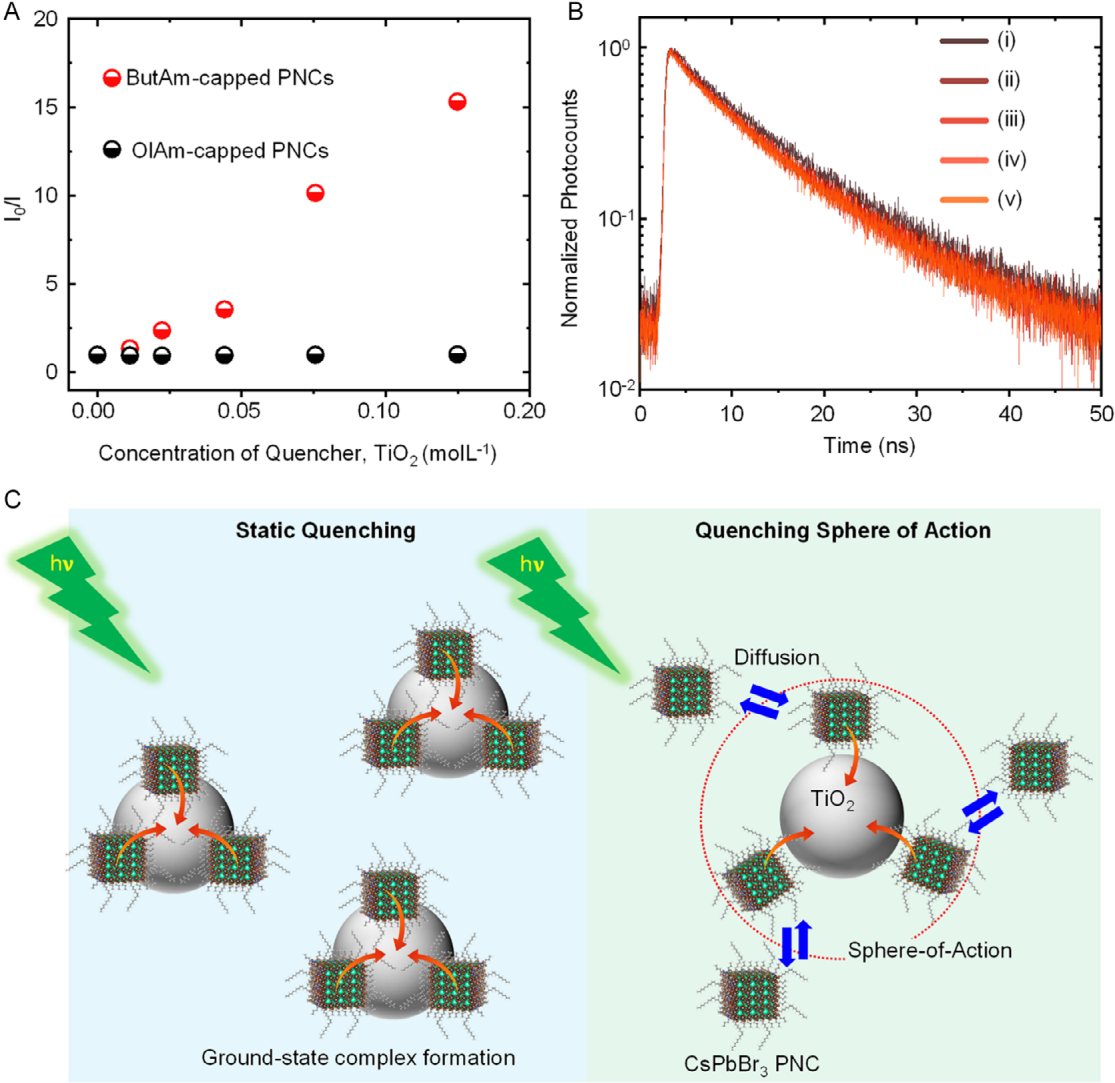
A) Stern–Volmer plots for OlAm‐ and ButAm‐capped CsPbBr_3_ PNC colloidal solutions in the presence of TiO_2_ as the quencher. B) PL decay profiles of ButAm‐capped CsPbBr_3_ PNC colloidal solution obtained after adding (i) 0 μL, (ii) 20 μL, (iii) 40 μL, (iv) 80 μL, and (vi) 240 μL of TiO_2_ NP suspension in toluene. C) Mechanism of PL quenching in CsPbBr_3_ PNCs by TiO_2_ NPs in solution.

To comprehend the mechanism of PL quenching in solution, we plotted Stern–Volmer plots^[^ ^27^ ^]^ for the OlAm‐ and ButAm‐capped CsPbBr_3_ PNCs at different concentrations of TiO_2_ as a quencher by taking the PL intensity values from Figure 4. The results are shown in **Figure** **5**A. The Stern–Volmer plot for OlAm‐capped PNCs exhibits a flat line (zero slope), confirming no PL quenching effect from TiO_2_. Conversely, the Stern–Volmer plot for ButAm‐capped CsPbBr_3_ PNC solution shows a nearly linear trend at lower concentrations, curving toward the *y*‐axis at higher concentrations. This behavior of the Stern–Volmer plot suggests a combination of static and dynamic PL quenching.^[^ ^27^ ^]^ In static PL quenching, the quencher forms a nonluminescent complex with the light‐emitting species before it is excited. In dynamic PL quenching, the quencher collides with the light‐emitting species in its excited state, preventing it from emitting light. In both cases, the Stern–Volmer plot yields a straight line with a slope. The distinguishing factor between these PL quenching mechanisms is the PL lifetimes of the light‐emitting species at varying quencher concentrations. For static quenching, PL lifetime remains constant regardless of quencher concentration. Conversely, in dynamic quenching, PL lifetime decreases as quencher concentration increases.^[^ ^27^ ^]^ In our study, the PL lifetime of ButAm‐capped CsPbBr_3_ PNCs remained constant across all TiO_2_ concentrations, as depicted in Figure 5B. This contradicts the nonlinear behavior observed in the Stern–Volmer plot in our case if we assume a prevalence of mixed static and dynamic quenching. In simple terms, we expected the PL lifetime to decrease as the quenching shifted from static to dynamic, but this was not observed in the PL decays. To better understand this, we looked beyond the traditional Stern–Volmer plot and considered deviations, incorporating mechanisms involving static quenching and quenching by the sphere of action,^[^ ^27^, ^28^ ^]^ as shown in Figure 5C.

During the static quenching, we assume a ground‐state complex formation by the adsorption of ButAm‐capped CsPbBr_3_ PNCs onto the surface of TiO_2_ (Figure 5C). The short‐chain butylamine ligand and the reduced concentration of oleic acid ligand on the surface of PNCs facilitated this adsorption. For the upward bending of the Stern–Volmer plot at the higher concentrations of TiO_2_, we propose PL quenching by the sphere of action mechanism in addition to the static quenching. Figure 5C shows quenching sphere of action mechanism where CsPbBr_3_ PNC and TiO_2_ do not form a stable ground‐state complex. Instead, an apparent static quenching results within a volume where the PNC emitter and TiO_2_ quencher are very close to each other at the time of excitation. Within this sphere of action, there exists a high probability that the PL quenching occurs before the PNC emitter and the TiO_2_ quencher diffuse apart. Further, the probability of PL quenching increases with increasing the concentration of the quencher. On the other hand, the presence of long‐chain oleylamine ligands and a higher concentration of oleic acid on the surface of as‐synthesized CsPbBr_3_ PNCs in conjunction with solvent intervention prevented their interaction with TiO_2_, resulting in no static or dynamic PL quenching. In line with our findings, recently, Vinçon et al. reported the interaction of metal salt BiBr_3_ with lecithin‐capped CsPbBr_3_ QDs through the sphere of action model which quenches PL in QDs but without significantly changing the PL lifetime.^[^ ^28^ ^]^ While they attribute the PL quenching in CsPbBr_3_ QDs by the controlled addition of Bi defects to the QD surface, we discuss PL quenching in ButAm‐capped CsPbBr_3_ PNCs by TiO_2_ NPs in solution in terms of donor–acceptor interactions leading to the electron transfer. Further, it is important to consider the artifact caused by the inner filtering effect which may result in PL quenching.^[^ ^29^ ^]^ Here, at a fairly high concentration of quencher that is added to the emitter in solution, the excitation light might get blocked by scattering or absorption by the quencher before reaching the emitter. This can also result in a lowering of PL intensity. In our case, such an inner filtering effect is trivial because we do not observe any decrease in PL intensity by adding the same concentration/amount of TiO_2_ to the OlAm‐capped CsPbBr_3_ PNC solution as in the case of ButAm‐capped ones. The exclusion of the inner filtering effect is further supported by the absorption spectra in Figure S6, Supporting Information, whose features are consistent over the range of TiO_2_ suspension added to the PNC solution.

In solution, we observed ligand chain length‐dependent PL quenching which is indicative of potential donor–acceptor interactions that could facilitate charge transfer between CsPbBr_3_ PNCs and TiO_2_ NPs. ButAm‐capped CsPbBr_3_ PNCs showed enhanced PL quenching and hence efficient electron transfer to TiO_2_ as compared with OlAm‐capped PNCs when photoexcited. However, the unchanged PL decay profiles regardless of the TiO_2_ NP concentration in the solution limited us from discussing the influence of donor–acceptor interactions on exciton recombination dynamics. Therefore, we investigated the CsPbBr_3_–TiO_2_ donor–acceptor system in its solid state with different chain length ligands on the PNC surface using a confocal fluorescence lifetime imaging microscopy (FLIM) or a time‐resolved PL spectroscopy. Further, to unveil the influence of TiO_2_ microstructure on electron transfer in a solid state we studied two systems, one involved a film formed by depositing a mixture of TiO_2_ NPs and OlAm‐ or ButAm‐capped CsPbBr_3_ PNC solution on a glass slide and the other was OlAm‐ or ButAm‐capped CsPbBr_3_ PNC layers deposited on the top of an anodically grown TiO_2_ NT array on a TiO_2_ substrate. The deposition in either of the cases was performed by spin coating followed by drying under argon. Contrary to the solution state, the samples in the solid state, regardless of ligand chain length, showed rapid PL decay in the presence of TiO_2_, indicating accelerated exciton recombination due to enhanced donor–acceptor interaction.^[^ ^30^ ^]^ The formation of a donor–acceptor interface between CsPbBr_3_ PNCs and TiO_2_ NPs in the solid state is observed on a TEM grid, which is shown in Figure S4B, Supporting Information. Previously, we demonstrated PL quenching revealed by the decrease in PL lifetimes across the heterojunction film formed by OlAm‐capped FAPbBr_3_ or CsPbBr_3_ PNCs with TiO_2_ and attributed the effect to carrier diffusion‐controlled electron transfer from PNCs to TiO_2_ at the interface.^[^ ^31^ ^]^ However, our previous study did not shed light on the influence of surface ligands on electron transfer in the solution or the film state, while, in this study, we show ligand chain length‐dependent donor–acceptor interactions between CsPbBr_3_ PNCs and TiO_2_ in solution and films and discuss different mechanisms involved therein.

**Figure 6:**
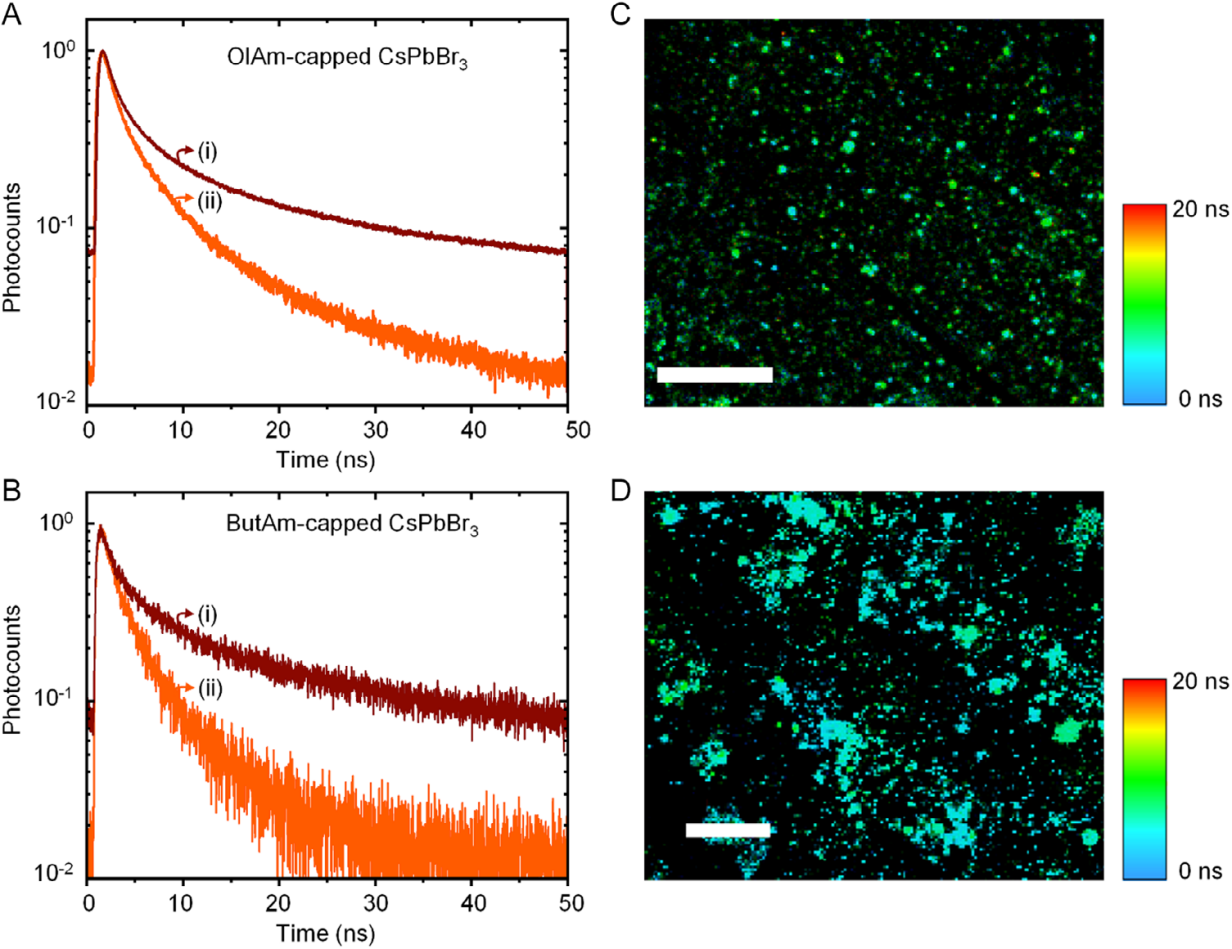
A,B) PL decay profiles of OlAm‐capped (A) and ButAm‐capped (B) CsPbBr_3_ PNC films before (i) and after (ii) mixing PNCs with TiO_2_. C,D) FLIM images of TiO_2_ mixed OlAm‐capped (C) and ButAm‐capped (D) CsPbBr_3_ films. The scale bars are 25 and 100 μm in (C,D), respectively.

The FLIM images for OlAm‐ and ButAm‐capped CsPbBr_3_ PNCs mixed with TiO_2_ NPs are shown in **Figure** **6**C,D, respectively, and the corresponding decay profiles are presented in Figure 6A,B. The OlAm‐ and ButAm‐capped CsPbBr_3_ PNC films without TiO_2_ show comparable average PL lifetime values of about 14 ns. Interestingly, when PNCs were mixed with TiO_2_ NPs to form films, the average PL lifetime decreased, especially for ButAm‐capped CsPbBr_3_ PNCs (≈6 ns), compared to OlAm‐capped PNCs (≈9 ns). This decrease suggested that the donor–acceptor interactions occurred in the film state for both OlAm‐ and ButAm‐capped CsPbBr_3_ PNCs, and this interaction probably led to an enhanced electron transfer for PNCs with short‐chain butylamine ligands. Furthermore, the contradicting results for PL quenching that occurs in the solid film but not in solution for OlAm‐capped PNCs suggest that, in the solution state, not only the ligand chain length but also the solvent interferes with the donor–acceptor interactions.

**Figure 7:**
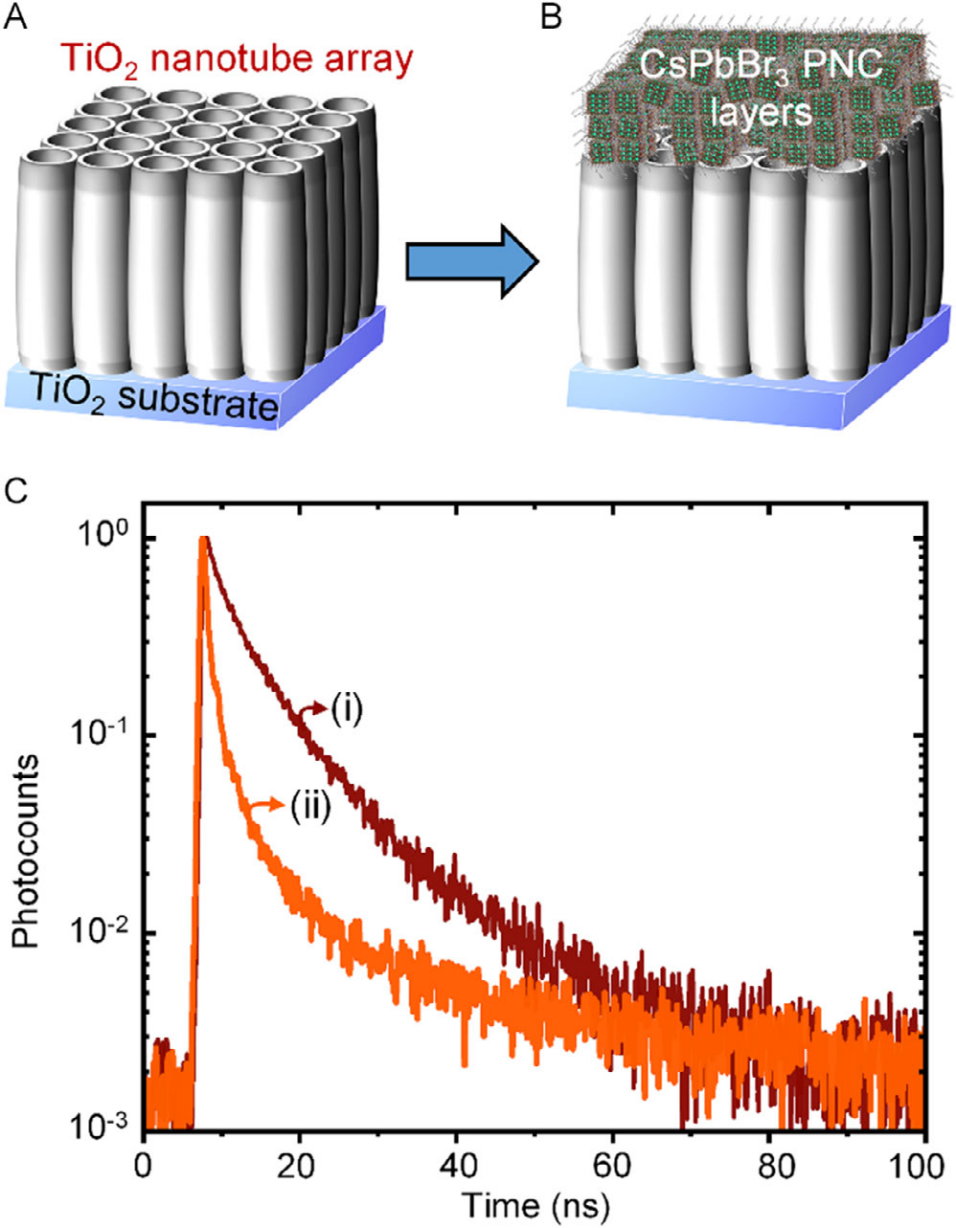
A,B) Scheme showing anodically grown TiO_2_ NT array on a TiO_2_ substrate (A) without CsPbBr_3_ PNCs and (B) with CsPbBr_3_ PNCs deposited on the top. C) PL decay profiles of ButAm‐capped CsPbBr_3_ PNCs deposited on (i) a glass coverslip and (ii) the top of the TiO_2_ NT array.

**Figure** **7**A,B shows schematic representations of the TiO_2_ NT array before and after the CsPbBr_3_ PNC deposition. The corresponding scanning electron microscopic (SEM) images are provided in Figure S7, Supporting Information. Additionally, Figure 7C shows the PL decay profiles for ButAm‐capped CsPbBr_3_ PNCs deposited on the glass slides and on top of the TiO_2_ NT array. The PL decay is rapid for PNCs deposited on top of the TiO_2_ NT array compared to those on a bare glass substrate, aligning with the trend discussed earlier. To further analyze this, we compared the PL lifetime components (*τ*_*i*_), corresponding fractional contribution (*f*_*i*_) of each decay time to the steady‐state intensity, and the average *τ*, obtained by fitting the PL decays with triexponential decay function, for these two systems: CsPbBr_3_ PNC–TiO_2_ NP film and CsPbBr_3_ PNC–TiO_2_ NT arrays. This comparison also included CsPbBr_3_ PNC films without TiO_2_. The results are summarized in **Table** **1**. For PNC films without TiO_2_, the average PL lifetime is largely contributed by the longest component *τ*_3_ which is 19 ns (66%) for OlAm‐capped CsPbBr_3_ PNCs and 18 ns (72%) for ButAm‐capped CsPbBr_3_ PNCs. On the other hand, the shortest component *τ*_1_, which is 1.18 ns for OlAm‐capped CsPbBr_3_ PNCs and 1.10 for ButAm‐capped ones, contributes less than 10% to the average PL lifetime. Additionally, the intermediate component *τ*_2_ (4.2 ns for OlAm‐capped and 3.8 ns for ButAm‐capped CsPbBr_3_ PNCs) contributes more than 20% to the average PL lifetime. Earlier reports suggest that *τ*_1_ is associated with the trapping of charge carriers in defect‐related shallow or deep trap states.^[^ ^32^ ^]^ Charge carriers quickly transition from the band edge to these trap states, depleting the concentration of carriers at the band edge and causing nonradiative recombination of excitons due to minimal detrapping.^[^ ^33^ ^]^ On the other hand, *τ*_2_ is influenced by exciton recombination at the band edge involving both radiative and nonradiative processes, as well as charge carrier trapping in defect‐related trap states. At this stage, the shallow traps are not fully saturated with charge carriers, affecting the trapping and detrapping rates. Despite a reduced trapping rate, the detrapping rate remains inefficient, resulting in a PL lifetime only slightly longer than *τ*_1_.^[^ ^33^ ^]^ The longest component *τ*_3_ correlates with radiative recombination at the band edge, particularly when shallow trap states are nearly saturated with charge carriers. This leads to a momentary trapping and subsequent detrapping of charge carriers, slowing down the radiative recombination of excitons at the band edge.^[^ ^33^, ^34^ ^]^ A significantly long and dominant *τ*_3_ in both film samples without TiO_2_ NPs indicates that excitons primarily recombine radiatively, consistent with the high absolute PL QY values observed for these samples.

**Table 1:** PL lifetime components (*τ*_*i*_), corresponding fractional contribution (*f*_*i*_) of each decay time to the steady‐state intensity, and intensity‐weighted average PL lifetimes (*τ*) obtained from multiexponential fitting of PL decay profiles of OlAm‐ and ButAm‐capped CsPbBr_3_ PNC film, CsPbBr_3_–TiO_2_ film, and CsPbBr_3_ PNCs deposited on the top of a TiO_2_ NT array

	*τ*_1_ [ns]	*f*_1_	*τ*_2_ [ns]	*f*_2_	*τ*_3_ [ns]	*f*_3_	Average *τ* [ns]
OlAm–CsPbBr_3_ PNC film	1.18	0.099	4.20	0.241	19.00	0.660	13.67
ButAm–CsPbBr_3_ PNC film	1.10	0.062	3.80	0.215	18.00	0.723	13.90
OlAm–CsPbBr_3_ PNC + TiO_2_ NP film	1.50	0.232	5.10	0.435	19.00	0.333	8.89
ButAm–CsPbBr_3_ PNC + TiO_2_ NP film	1.40	0.221	3.60	0.454	12.00	0.325	5.84
ButAm–CsPbBr_3_ PNCs on TiO_2_ NT array	0.35	0.230	1.83	0.434	9.33	0.332	3.97

We observed a distinct shift in the contribution of the radiative and nonradiative exciton recombination processes on the overall PL lifetime of CsPbBr_3_ PNCs in their films in the absence or presence of TiO_2_ (Table 1). Specifically, in the OlAm‐capped CsPbBr_3_ PNC–TiO_2_ NP film, *τ*_1_, *τ*_2_, and *τ*_3_ values remained unchanged compared to the film without TiO_2_. However, their relative contributions to the average PL lifetime shifted from the longest component *τ*_3_ (from 66% to 33%) to the shortest *τ*_1_ (from 10% to 23%) and intermediate *τ*_2_ (from 24% to 44%). This suggests that the introduction of TiO_2_ NPs as electron acceptors does not create fresh nonradiative decay paths for excitons in OlAm‐capped CsPbBr_3_ PNCs within the film. Instead, TiO_2_ NPs likely favor the nonradiative recombination affecting the overall rate at which excitons recombine, possibly through the modification of trap states. Further study is required to completely understand the influence of charge acceptors on the modification of existing trap states in metal halide perovskites. On the other hand, we observed a shift from the contribution of *τ*_3_ (from 72% to 33%) to *τ*_1_ (from 6% to 22%) and *τ*_2_ (from 22% to 45%) in the average PL lifetime, along with a decrease in *τ*_3_ value from 18 ns to 12 ns in the case of ButAm‐capped CsPbBr_3_ PNC–TiO_2_ NP film. This implies that TiO_2_ NPs not only favor the nonradiative exciton recombination in ButAm‐capped CsPbBr_3_ PNCs but also impact the radiative recombination by altering the rate of electron detrapping from the shallow trap states. We propose that in ButAm‐capped CsPbBr_3_ PNCs, electron transfer from shallow trap states to TiO_2_ NPs competes with the detrapping of electrons to the band edge. The former process takes precedence, resulting in faster radiative recombination of the untrapped excitons at the band edge. In the case of ButAm‐capped CsPbBr_3_ PNCs on the TiO_2_ NT array, the alteration in the contributions of PL lifetime components *τ*_1_, *τ*_2_, and *τ*_3_ to the average PL lifetime aligns with that seen in PNC–TiO_2_ NP films (Table 1). However, the values of all three PL lifetime components decrease notably to 0.35 ns for *τ*_1_, 1.83 ns for *τ*_2_, and 9 ns for *τ*_3_. Particularly, the *τ*_1_ decrease is substantial compared to PNC films with TiO_2_ NPs. These findings highlight that, in the ButAm‐capped CsPbBr_3_ PNC–TiO_2_ NT array system, TiO_2_ not only impacts existing recombination processes but also creates new nonradiative decay paths for excitons. This can be attributed to the efficient charge transfer from CsPbBr_3_ PNCs to TiO_2_ NTs.

## 3 Conclusion

We explored the impact of altering ligand chain lengths on CsPbBr_3_ PNCs by employing a postsynthesis ligand exchange strategy. This method allowed us to replace long‐chain ligands with shorter ones, enabling the study of distance‐dependent photophysical interactions. The length of the ligand chain proved to be a critical factor affecting donor–acceptor interactions, influencing PL quenching and electron transfer within CsPbBr_3_ PNCs. Our investigation focused on understanding these interactions in the CsPbBr_3_–TiO_2_ system, both in solution and film states. In the solution phase, we observed enhanced donor–acceptor interactions for PNCs with short‐chain butylamine ligands, leading to a mixed PL quenching interaction encompassing static quenching and quenching sphere of action. Conversely, CsPbBr_3_ PNCs with oleylamine ligands in solution showed no notable changes in PL properties in the presence of TiO_2_ NPs, suggesting poor donor–acceptor interactions due to the presence of long‐chain ligands on the PNC surface. In dried films, both long‐ and short‐chain ligand‐capped CsPbBr_3_ PNCs exhibited rapid PL decay in the presence of TiO_2_, implying accelerated exciton recombination. Intriguingly, solid‐state PL quenching for long‐chain ligand‐capped PNCs contradicted the solution state, highlighting the influence of the solvent on donor–acceptor interactions. Additionally, the exciton recombination dynamics in PNCs are affected more by ligand chain length in the presence of TiO_2_ NTs compared to TiO_2_ NPs, illustrating the effect of the TiO_2_ microstructure on charge transfer. In summary, our study sheds light on the intricate interplay between TiO_2_ and the ligands of CsPbBr_3_ PNCs, providing crucial insights for their application in optoelectronics.

## 4 Experimental Section

4.1

4.1.1

##### Materials

All the materials were ordered from Sigma–Aldrich unless otherwise stated and used without any further purification, which includes: lead (II) bromide (PbBr_2_, 98+%), cesium acetate (CH_3_COOCs, >98%), oleic acid (90%), oleylamine (80–90%), butylamine (99+%), octadecene (90%), and toluene (99.5%). Titanium oxide (TiO_2_) NP powder was obtained from Kemira Global, Finland.

##### Synthesis of CsPbBr_3_ PNCs

CsPbBr_3_ PNCs were synthesized using a hot injection method reported previously with slight modifications.^[5a]^ During the synthesis, CH_3_COOCs (0.4 mmol, 77 mg) and oleic acid (1.58 mmol, 500 μL) were added to octadecene (1 mL) in a three‐neck flask, and the mixture was heated to 120 °C under an inert atmosphere of argon. After the temperature was stabilized, the mixture was switched to the vacuum condition for 1 h for drying. In parallel, PbBr_2_ (0.4 mmol, 146 mg), oleic acid (4 mmol, 1.262 mL), and oleylamine (4 mmol, 1.316 mL) were added to octadecene (25 mL) in a separate three‐neck flask. The mixture was heated to 120 °C under an argon atmosphere and after the temperature was stabilized, the mixture was switched to the vacuum condition for 1 h for drying. Once the precursors were completely dissolved in octadecene, a clear solution was obtained. Afterward, the temperature of PbBr_2_ precursor solution was increased to 155 °C, and 1 mL of cesium precursor was injected into it, resulting in the formation of a green precipitate of CsPbBr_3_ PNCs. The reaction was quenched by placing the reaction flask in an ice‐water bath after 2 s of the reaction time. The crude CsPbBr_3_ PNCs were collected by centrifugation at 10 000 rpm for 10 min, and the precipitate was suspended in toluene. The resulting colloidal solution was centrifuged again at 6000 rpm for 10 min to collect the final CsPbBr_3_ PNC precipitate which was then resuspended in 6 mL of toluene for further studies.

##### Ligand Exchange in CsPbBr_3_ PNCs

A postsynthesis ligand exchange was performed under ambient conditions to exchange the oleylamine ligand with butylamine ligand on the surface of CsPbBr_3_ PNCs. A 50 μL of OlAm‐capped CsPbBr_3_ PNCs (6 μmol) in toluene was diluted to 3 mL. Also, 2 μL of butylamine (20 μmol) were added to a 500 μL toluene. The butylamine solution was then added to the CsPbBr_3_ PNC solution and the mixture was stirred at 500 rpm for 10 min under air atmosphere and at room temperature. This was followed by washing of excess ligands using acetonitrile at 5000 rpm for 5 min. The obtained precipitate was resuspended in toluene.

##### Growth of TiO_2_ NT Arrays

TiO_2_ NT arrays were produced by anodic oxidation of commercial purity, Grade 2 ASTM, titanium sheets that were 0.5 mm thick. From the sheets, 1.5 × 1.5 cm^2^ size slabs were cut and polished with P600 SiC paper. The TiO_2_ slabs were then subjected to sonication in ethanol to remove surface contaminations and anodized in an ethylene glycol solution containing 0.2 m of NH_4_F and 2 m of distilled water. A cell voltage of 45 V was reached by a linear sweep in 2 min and maintained for 30 min in the potentiostatic conditions, as reported in previous studies.^[^ ^35^ ^]^ At the end of the anodizing procedure, TiO_2_ slabs were carefully rinsed with distilled water and subjected to annealing at 500 °C for 2 h to obtain oxide crystallization in the anatase phase.

##### Preparation of Thin Film Samples

Thin films of OlAm‐ and ButAm‐capped CsPbBr_3_ PNCs with or without TiO_2_ NP powder were spin‐coated onto 18 mm × 18 mm glass coverslips (Menzel‐Glaser) at 1000 rpm for 2 s. Before deposition, the glass coverslips were treated ultrasonically with isopropyl alcohol and acetone, respectively, followed by drying using an argon gun. To avoid and minimize the effect of aging and degradation, freshly synthesized PNCs were used, and the measurements were done immediately after the ligand exchange to ensure comparability.

Similarly, freshly prepared OlAm‐ or ButAm‐capped CsPbBr_3_ PNCs were deposited onto the anodically grown TiO_2_ NT array by spin coating the colloidal solutions of PNCs at 1000 rpm for 2 s, and the measurements were carried out after drying under the argon atmosphere.

##### Characterizations

FTIR scans were run by using Spectrum Two FT‐IR Spectrometer from Perkin Elmer. The measurements were performed by drying the PNC colloidal solutions on a diamond‐attenuated total reflection unit in a range from 500 to 4000 cm^−1^.

STEM images of CsPbBr_3_ PNCs were acquired on a Jeol ARM20CF NeoARM electron microscope at 200 kV acceleration voltage. The samples were prepared by drop‐casting the colloidal solution on the carbon‐coated TEM grids. SEM images of anodically grown TiO_2_ NTs with CsPbBr_3_ PNC deposition were recorded on a Gemini Supra 25 field‐emission SEM from Zeiss at an acceleration voltage of 10 kV. Similarly, the SEM images of bare anodically grown TiO_2_ NTs were recorded on a Gemini SEM from Zeiss at an acceleration voltage of 5 kV. Powder XRD of the samples was measured using an Aeris X‐ray diffractometer from Malvern Panalytical (Cu K*α*1, 1.5406 Å). The samples were prepared by drop‐casting the PNC colloidal solution on a low‐background silica disc.

UV–vis absorption spectra of CsPbBr_3_ PNCs and TiO_2_ NPs were recorded in a quartz cuvette using a Lambda 1050 + UV–vis–NIR spectrometer from Perkin Elmer. Steady‐state and time‐resolved PL were recorded on Spectrofluorometer FS5 from Edinburg Instrument. For the time‐resolved PL measurements, a picosecond laser with 375 nm excitation wavelength was used and the data acquisition was made using the technique of TCSPC. The decay profiles were tail‐fitted with a triexponential function R (t)=A1exp(−tτ1)+A2exp(−tτ2)+A3exp(−tτ3) and the intensity‐weighted average PL lifetime was calculated using the formula, $\tau_{\text{av}}=\frac{A_{1}\tau_{1}^{2}{+A}_{2}\tau_{2}^{2}{+A}_{3}\tau_{3}^{2}}{A_{1}\tau_{1}{+A}_{2}\tau_{2}{+A}_{3}\tau_{3}}$. Also, the fractional contribution of each decay time to the steady‐state intensity was calculated using the formula, $f_{i}=\;\frac{A_{i}\tau_{i}}{A_{1}\tau_{1}{+A}_{2}\tau_{2}{+A}_{3}\tau_{3}}$.

PL QYs of the CsPbBr_3_ PNCs were measured using an absolute method by directly exciting the PNC solution as the sample and the toluene as the reference in an SC‐30 integrating sphere module fitted to a Spectrofluorometer FS5 from Edinburg Instrument. During the measurement, the excitation slit was set to 3 nm, and the emission slit was adjusted to obtain a signal level of 1 × 10^6^ cps. A wavelength step size of 0.1 nm and an integration time of 0.2 s were used. The calculation of absolute PL QY is based on the formula, $\text{PL QY}=\frac{E_{\text{sample}}-E_{\text{ref}\text{.}}}{S_{\text{ref}\text{.}}-S_{\text{sample}}}$, where *E*_sample_ and *E*_ref_ are the integrals at the emission region for the sample and the reference, respectively, and *S*_sample_ and *S*_ref_ are the integrals at the excitation scatter region for the sample and the reference, respectively. The selection and calculation of integrals from the emission and excitation scattering region and the calculation of absolute PL QY were performed using the FLUORACLE software from the Edinburg Instrument. For steady‐state PL spectra and absolute PL QY measurements, the samples were excited at 450 nm.

FLIM measurements were performed in the confocal configuration in an ambient atmosphere on a MicroTime200 fluorescence microscope from PicoQuant equipped with 440 nm picosecond laser, 60× objective, PMA Hybrid single photon detector, and PicoHarp 300 TCSPC module. The excitation spot size was estimated at 550 nm (FWHM).

## Conflict of Interest

The authors declare no conflict of interest.

## Supporting information

Supplementary Material

## Data Availability

The data that support the findings of this study are available from the corresponding author upon reasonable request.
