# Supplementary material for: Tailoring Charge Donor–Acceptor Interaction in CsPbBr3 Perovskite Nanocrystals through Ligand Exchange
Source: Small Sci. 2024 Mar 26;4(5):2300348. doi: 10.1002/smsc.202300348 (PMC11935244; doi:10.1002/smsc.202300348)
Supplement: Supplementary file 1 — Supplementary Material [file SMSC-4-2300348-s001.pdf]

Tailoring Charge Donor-Acceptor Interaction in $CsPbBr_3$ Perovskite Nanocrystals through Ligand Exchange

Syed Abdul Basit Shah[1], Sushant Ghimire[2,3*], Rostyslav Lesyuk[2,4], Maria Vittoria Diamanti[5], Vanni Lughi[1] and Christian Klinke[2,6,7*]

[1]Department of Engineering and Architecture, Università degli studi di Trieste, Trieste, Italy;

[2]Institute of Physics, University of Rostock, 18059 Rostock, Germany;

[3]Chair for Photonics and Optoelectronics, Nano-Institute Munich and Department of Physics, Ludwig-Maximilians-Universität (LMU), 80539 Munich, Germany;

[4]Pidstryhach Institute for Applied Problems of Mechanics and Mathematics of NAS of Ukraine, Lviv, Ukraine;

[5]Department of Chemistry, Materials and Chemical Engineering "Giulio Natta" Politecnico di Milano, Italy;

[6]Department of Chemistry, Swansea University, Swansea, United Kingdom;

[7]Department "Life, Light & Matter", University of Rostock, 18059 Rostock, Germany.

*Corresponding Authors: christian.klinke@uni-rostock.de; sushant.ghimire@lmu.de

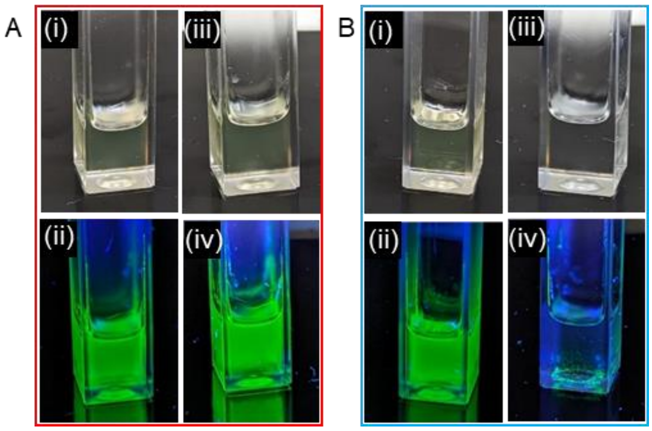

Figure S1. Stability of $CsPbBr_3$ PNCs after ligand-exchange with different amounts of butylamine. (A) Cuvette images of $CsPbBr_3$ PNC solution (i/ii) immediately and (iii/iv) two hours after the treatment with 2 μL of butylamine. (B) Cuvette images of $CsPbBr_3$ PNC solution (i/ii) immediately and (iii/iv) two hours after the treatment with 10 μL of butylamine. (i/iii) and (ii/iv) in A and B are the images under room light and UV light, respectively.

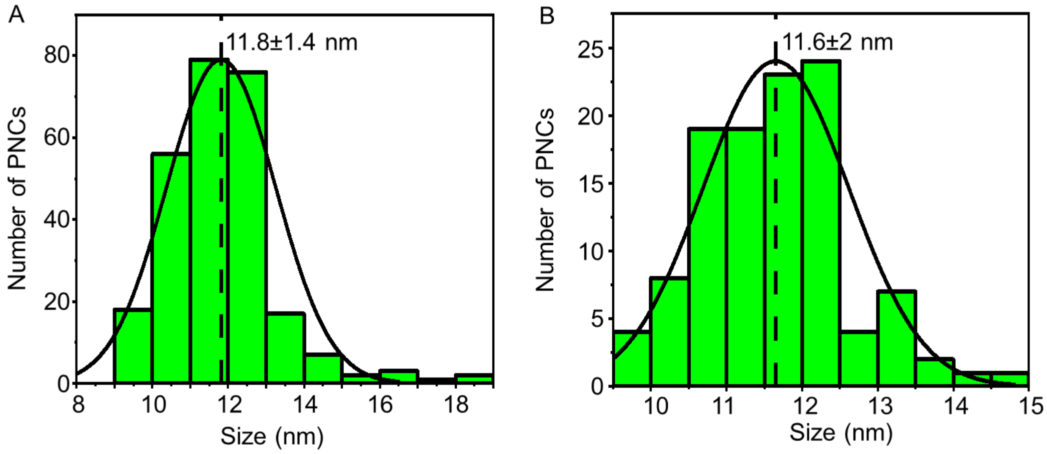

Figure S2. Size distribution histograms for (A) oleylamine (OlAm)-capped and (B) butylamine (ButAm)-capped $CsPbBr_3$ perovskite nanocrystals (PNCs).

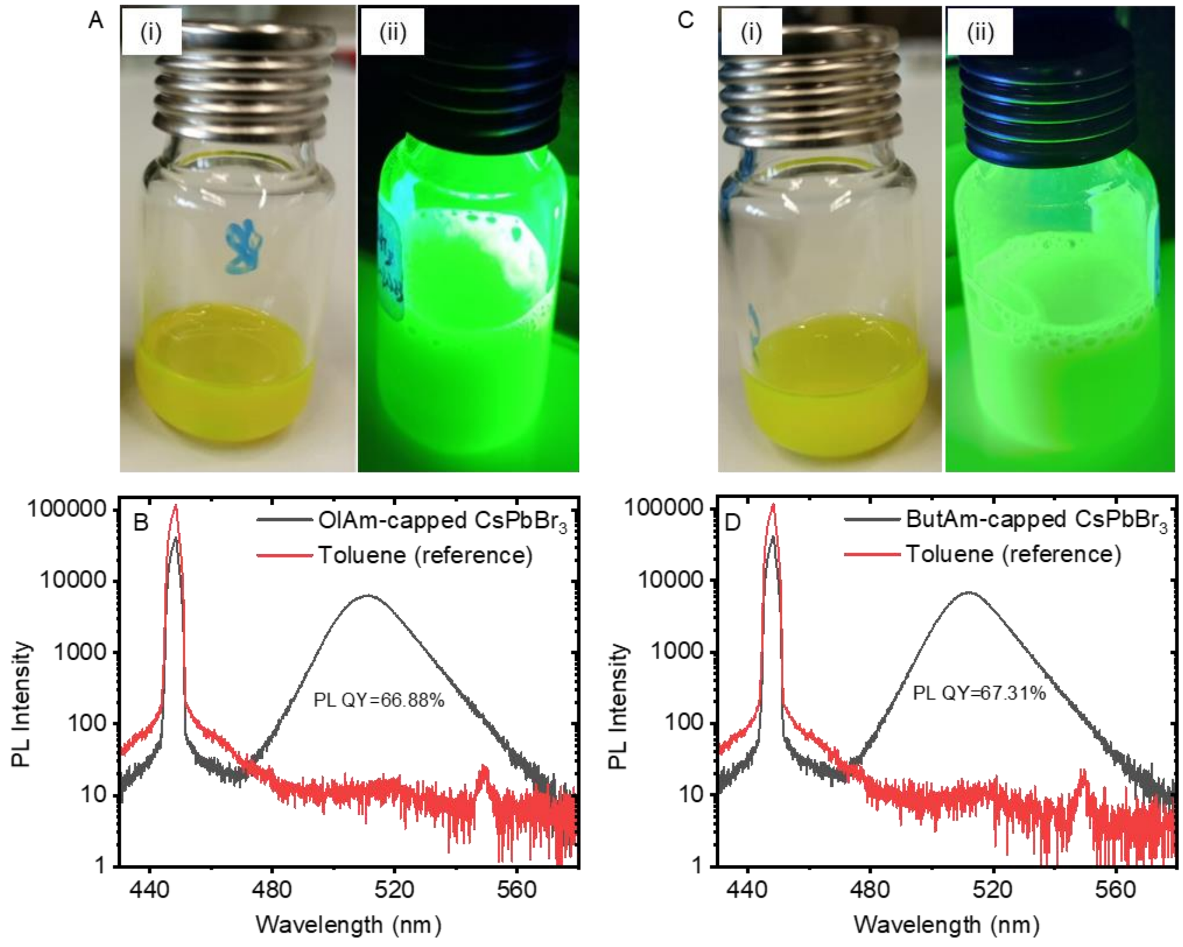

Figure S3. (A, B) Colloidal solutions of (A) OlAm-capped and (B) ButAm-capped $CsPbBr_3$ PNCs (i) at room light and (ii) under 365 nm UV lamp excitation. (B, C) Photoluminescence (PL) spectra recorded with an integrating sphere in a PL spectrometer for the absolute PL quantum yield (QY) measurements of (B) OlAm-capped and (D) ButAm-capped $CsPbBr_3$ PNC colloidal solutions. Toluene was used as the reference.

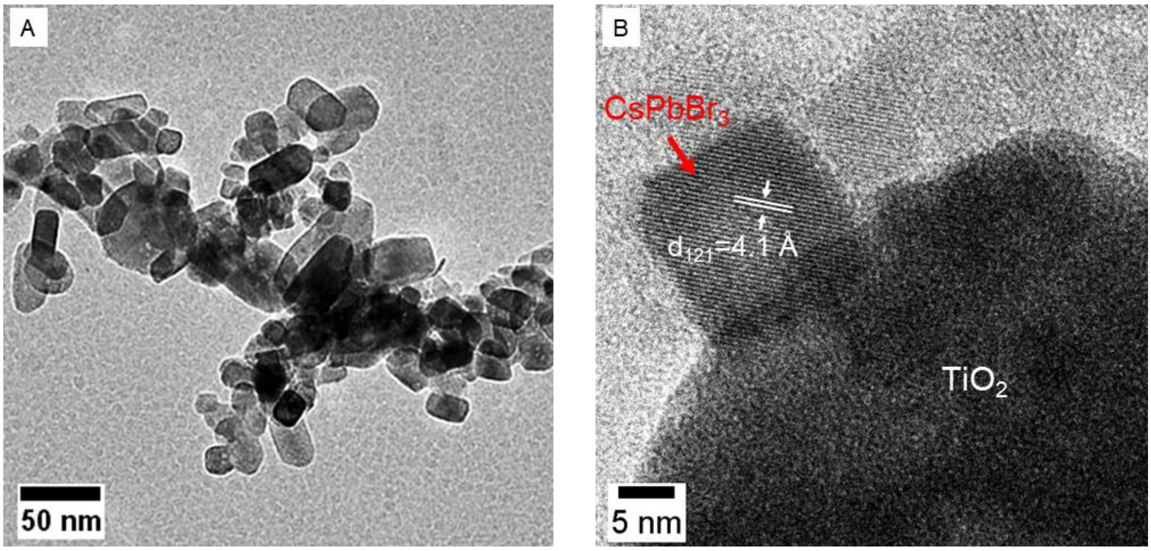

Figure S4. Transmission electron microscopy (TEM) images of (A) TiO_2 nanoparticles and (B) a $CsPbBr_3$ PNC on the TiO_2 nanoparticle showing a donor-acceptor interface. A lattice fringe of 4.1 Å in $CsPbBr_3$ PNC corresponds to the (121) plane of an orthorhombic crystal structure of the bulk $CsPbBr_3$, as reported in ref. 1.

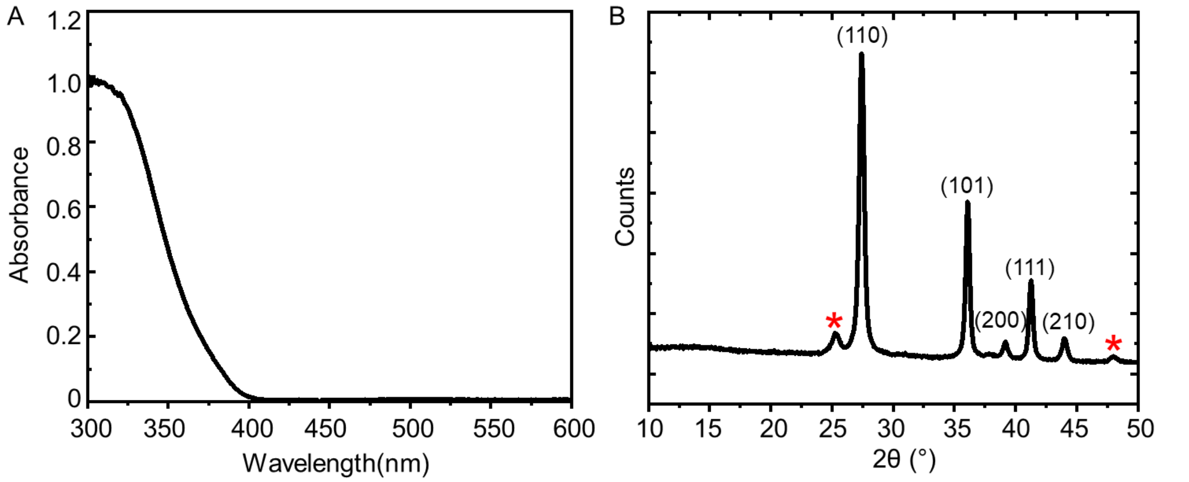

Figure S5. (A) UV-Visible absorption spectrum of TiO_2 nanoparticle suspension. (B) Powder X-ray diffraction (XRD) pattern of TiO_2 nanoparticles. The indexed XRD peaks are for Rutile phase, while the XRD peaks labelled with (*) are for the Anatase phase of TiO_2. The XRD peaks are analysed by comparing them with those from ref. 2.

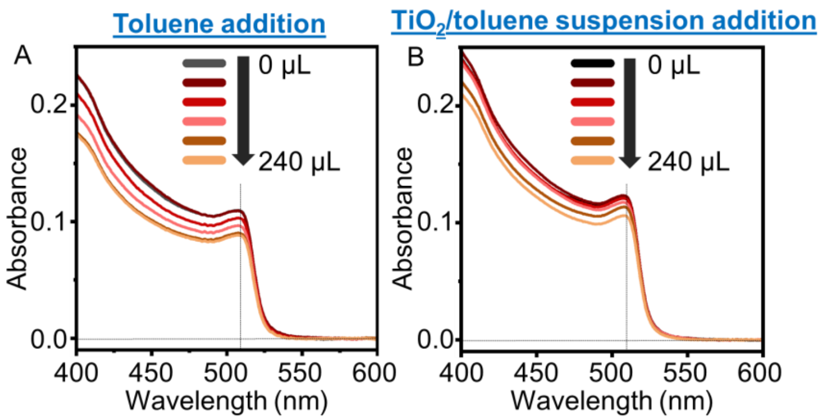

Figure S6. Absorption spectra of butylamine-capped $CsPbBr_3$ PNCs with the (A) addition of toluene and (B) TiO_2-suspended toluene at different volumes (indicated in the inset), indicating colloidal as well as material stability under dilution.

In Figure S6, we see only a gradual decrease in the absorption peak which is due to the dilution, but without the appearance of any addition bands in the absorption spectra in both the cases. This suggest that the PNCs are stable during the addition of TiO_2 or dilution. Further, we do not observe significant scattering at higher wavelength region, tailing at the absorption onset, or broadening of the absorption peak in the absorption spectra, suggesting that the colloidal stability is intact.

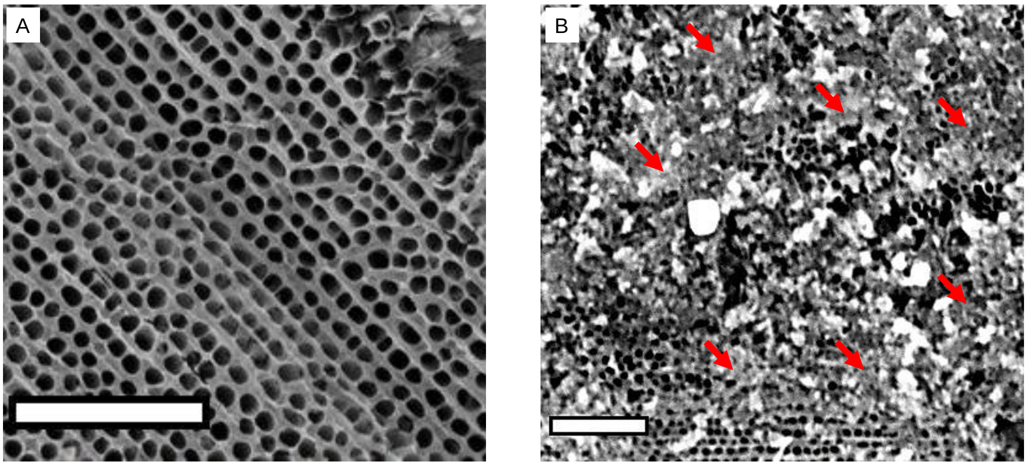

Figure S7. Scanning electron microscopy images of (A) anodically-grown bare TiO_2 nanotubes and (B) TiO_2 nanotubes with $CsPbBr_3$ PNCs deposited on the top. Some patches of deposited PNCs are indicated by red arrows. Scale bars in both images are 1 μm.

Dilution correction for the PL spectra of $CsPbBr_3$ PNCs in presence of quencher (TiO_2)

The addition of toluene causes a decrease in PL intensity of PNC solution due to the dilution effect. Therefore, to separate out the effect of dilution from the effect of charge transfer interaction on the PL intensity of PNC solution, two separate set of experiments were carried out. In the first experiment, TiO_2 suspended in toluene was added to PNC solution following a step wise manner as presented in the main text and the corresponding PL spectra were recorded. In the second set of experiments, similar volume of only toluene (but without TiO_2) was added in a step wise manner to the PNC solution to obtain a set of corresponding PL spectra. The data from both experiments were compared to subtract out the effect of toluene dilution.

We divide the PNC solution into 2 cuvettes (cuvette1 and cuvette2), each having equal volume. We take cuvette1, record the initial PL intensity (P_1) and add 20 µL (TiO_2/toluene suspension) to it, which causes a decrease in the PL intensity to P_1'. This decrease is ascribed to the combined effect of charge transfer and dilution.

Again, we take cuvette2, record the initial PL intensity (P_2) and add 20µL of pure toluene to it, causing decrease in PL intensity to P_2'. This decrease in PL intensity is now ascribed to only the dilution effect.

For the dilution correction on PL spectra, we carry out a two-step calculation:

$$\mathbf{(P_2 - P_2' / P_2) = Z} \quad \text{(dilution factor)}$$

$$\mathbf{P_1' + (P_1 \times Z)} \quad = \textit{Corrected PL intensity (without the dilution effect of solvent, toluene)}$$

The same process was repeated for all the subsequent step wise addition of TiO_2 suspension to the PNC solution.

References

1 C. C. Stoumpos, C. D. Malliakas, J. A. Peters, Z. Liu, M. Sebastian, J. Im, T. C. Chasapis, A. C. Wibowo, D. Y. Chung, A. J. Freeman, B. W. Wessels and M. G. Kanatzidis, *Cryst. Growth Des.*, 2013, **13**, 2722–2727.

2 K. K. Tehare, S. S. Bhande, S. U. Mutkule, F. J. Stadler, J.-P. Ao, R. S. Mane, X. Liu, *J. Alloys Compd.*, 2017, **704**, 187–192.
